# Unraveling the Role of Guanylate-Binding Proteins (GBPs) in Breast Cancer: A Comprehensive Literature Review and New Data on Prognosis in Breast Cancer Subtypes

**DOI:** 10.3390/cancers14112794

**Published:** 2022-06-04

**Authors:** Erin N. Hunt, Jonathan P. Kopacz, Deborah J. Vestal

**Affiliations:** Department of Biological Sciences, University of Toledo, Toledo, OH 43606, USA; erin.hunt@rockets.utoledo.edu (E.N.H.); jonathan.kopacz@rockets.utoledo.edu (J.P.K.)

**Keywords:** estrogen receptor (ER), guanylate-binding protein (GBP), GTPase, recurrence-free survival (RFS), overall survival (OS), progesterone receptor (PR), distant metastasis-free survival (DMFS), triple negative breast cancer (TNBC), interferon-γ (IFN-γ), signal transducer and activator of transcription 1 (STAT1)

## Abstract

**Simple Summary:**

Interferons are important in normal breast development, but also in the development and progression of breast cancers. Recently, three members of the Guanylate-Binding Protein (GBP) family of interferon-induced GTPases, GBP-1, GBP-2, and GBP-5, have been implicated to play roles in breast cancer. Both GBP-1 and GBP-5 are suggested to be potential drug targets in breast cancers, despite that there is no consensus on whether they are associated with better or worse prognoses. In fact, most of the literature related to GBPs in breast cancer suggests their expression correlates with improved prognoses. This manuscript will identify some of the reasons for this lack of consensus.

**Abstract:**

At least one member of the Guanylate-Binding Protein (GBP) family of large interferon-induced GTPases has been classified as both a marker of good prognosis and as a potential drug target to treat breast cancers. However, the activity of individual GBPs appears to not just be tumor cell type–specific but dependent on the growth factor and/or cytokine environment in which the tumor cells reside. To clarify what we do and do not know about GBPs in breast cancer, the current literature on GBP-1, GBP-2, and GBP-5 in breast cancer has been assembled. In addition, we have analyzed the role of each of these GBPs in predicting recurrence-free survival (RFS), overall survival (OS), and distance metastasis-free survival (DMFS) as single gene products in different subtypes of breast cancers. When a large cohort of breast cancers of all types and stages were examined, GBP-1 correlated with poor RFS. However, it was the only GBP to do so. When smaller cohorts of breast cancer subtypes grouped into ER+, ER+/HER2−, and HER2+ tumors were analyzed, none of the GBPs influenced RFS, OS, or DMSF as single agents. The exception is GBP-5, which correlated with improved RFS in HER2+ breast cancers. All three GBPs individually predicted improved RFS, OS, and DMSF in ER− breast cancers, regardless of the PR or HER2 status, and TNBCs.

## 1. Introduction

### Interferons and Breast Cancer

Cytokines are important in both normal breast development and the development and progression of breast cancers (reviewed in [1,2,3]). The cytokine IFN-γ is important in the development of cells of the immune system with anti-tumor activities and with immunoediting (reviewed in [1,2,3,4,5,6]). The role of IFN-γ and IFN-γ-response genes in cancer development is still incompletely understood. The evidence suggests both a pro- and anti-cancer role for IFN-γ and its response genes. This could be the reflection of differences in the cell type of the cancer and/or the stage of its development. This also suggests that IFN-γ and IFN-γ-response genes can function as double-edged swords in cancer.

STATs are important transcription factors in the signaling cascade of multiple cytokines, including IFN-γ (reviewed in [1,2]). Mice lacking functional STAT1, a transcription factor utilized by all three classes of interferons, develop breast cancer spontaneously [7]. These spontaneous tumors are ER+/PR+ luminal mammary carcinomas. Immunohistochemical (IHC) analysis of ER− versus ER+ breast tumors from patients showed that 37/83 or 45% of ER+ breast cancers had low or undetectable STAT1, while 17/78 or 22% of ER− breast cancers had low to undetectable STAT1 [7]. The observation that the STAT1 levels in normal breast tissue was significantly higher than in matched breast tumor samples suggested that its expression was lost during tumor progression. That STAT1 was uniformly higher in stromal tissue surrounding tumors than in the tumor cells themselves suggested that the regulation of STAT1 in tumor cells was independent of stromal cells [7]. The anti-tumor function of STAT1 could be restored by forced expression in breast cancer cells derived from STAT null mice and injected into null mice. This suggests that the anti-tumor activity of STAT1 is cell autonomous [7].

An anti-tumor role of STAT1 was confirmed by studying the highly metastatic murine breast cancer cell line 4T1.2 in STAT1 null BALB/c mice [8]. Orthotopic injection of 4T1.2 cells into the mammary fat pads of BALB/c mice demonstrates that lack of STAT1 in non-tumor cells results in significantly larger primary tumors and greater numbers of lung metastases. This suggests a non-cell autonomous anti-tumor role for STAT1 [8]. Consistent with this, STAT null mice have greater accumulation of both myeloid subtypes of myeloid derived suppressor cells (MDSC) than tumors grown in WT mice [8]. These cells are known to inhibit cytotoxic and helper T-cells with anti-tumor activity. In addition, the tumor cells in this environment express higher *mmp-9* and *cxcl1* than in WT mice [8]. Furthermore, the tumors in STAT1 null mice had increased CD31 positive cells compared to that in WT mice, indicating that, in the absence of STAT1 in the environment, the angiogenesis in the primary tumors was greater. GBP-1, an IFN-γ induced gene, inhibits angiogenesis [9,10]. The frequency of both CD4+ and CD8+ T cells was reduced in the STAT1 null mice compared to the WT mice [8]. This study suggests both a cell autonomous and non-cell autonomous role for STAT1 in cancer.

Interferons and their response genes were examined for possible roles in treatment or defense against different cancers, in large part due to their antiproliferative activity on tumor cells [11], and moreover, because they are regulators of the anti-tumor immune response [1,2,4,12]. Human mammary epithelial cells (MEC) were analyzed for the in vitro effect of IFN-γ treatment, which was accompanied by a block in G1 [11]. This block corresponds to an inhibition of the hyperphosphorylation of Rb, required for transition through the restriction point. Significantly, treatment of a panel of breast cancer cell lines demonstrated that 50% of the cell lines (3/6) failed to induce the expression of GBP-1 upon IFN-γ treatment, indicating a defect in IFN-γ signaling [11].

The role of individual cytokines and other proteins in the development and progression of cancer is exceedingly complicated. Thus, much work has been done to identify gene expression profiles that predict prognosis in breast cancers [13,14]. While many of these studies rely on gene expression arrays that do not distinguish between tumor cells and other cells of the tumor environment, some also use IHC to identify which cells within the tumor and surrounding environment express the identified genes [13,14]. In one such study, the goal was to identify immune function genes in which their expression would distinguish high from low risk of distant relapse in breast cancers, regardless of hormone receptor status [14]. In a retrospective study employing samples from eight patients with recurrence between 1 and 5 years and nine patients with at least 7 years recurrence-free, RNA was extracted and used to probe an Operon Human Genome Array-Ready with 25,100 genes. A total of 349 genes were identified between the two groups of patients, and 299 of those were upregulated in the recurrence-free patients. These included a cohort of genes involved in T-cell activity, B-cell activation, and antigen presentation. These genes did not overlap on heat maps comparing the two patient sample groups with *p* < 0.001 by students *t*-test. The genes that best separated the two groups were IGKC, GBP1, STAT1, IGLL5, and OCLN [14]. IHC confirmed the upregulation of STAT1 and GBP1 in the recurrence-free group. The STAT1 staining was strong in the infiltrating cells and the tumor cells of the recurrence-free group. It was expressed at lower levels and primarily in the infiltrating cells of the patients with early recurrence. GBP1 was expressed most robustly in the infiltrating cells but also in the tumor cells of the recurrence-free group. It was expressed weakly in the tumor cells of the samples with earlier recurrence [14].

The observation that a molecular signature correlated with NK cells and the 5 gene signature described above being predictive of longer relapse-free survival (RFS) confirms that an IFN-γ-driven gene signature is an important predictor of better outcome in breast cancers [13]. Since the tumor microenvironment plays a significant role in the prognosis of breast cancers, investigators explored whether combining an IFN-γ gene signature with an ECM gene signature would further refine the prediction of prognosis, specifically in high grade breast cancers [15]. The authors had previously identified a profile of ECM genes (ECM3) expressed by both tumor cells and stromal cells that correlated with higher relapse rate. In this study they added an IFN metagene signature to their analyses of high-grade breast cancers and found that the worst prognosis was observed where the tumor was positive for the ECM3 signature but negative for the IFN metagene signature [15]. These tumors were low in TILs, low in PD-1 or PD-L1, and had high levels of CD33-cells [15].

Recently, several manuscripts grouped TNBCs into subcategories based on microarray analyses [16,17,18]. One paper grouped the basal-like TNBCs into two subgroups: Basal-like immune suppressed (BLIS) and Basal-like immune activated (BLIA) [16]. Despite arising from the same histological cell type, the two subgroups had vastly different prognoses. BLIA had a 10-year survival of about 80% and BLIS had a 5-year survival of about 35%. The BLIS cluster does not express genes involved in B-cell, T-cell, and NK cell activation pathways. They also have minimal expression of genes involved in antigen presentation and other aspects of immune regulation. The BLIA subtype was characterized by the up-regulation of genes that control the regulation of the immune system, which includes genes involved in the activation of T-cells, B-cells, and NK cells and genes activated by STAT, in general suggesting an IFN-γ mediated gene expression pattern. The BLIA cluster had the highest disease-free survival rate of all TNBCs. GBP-5 was the 5th most robustly expressed gene in the BLIA subgroup. GBP-1 was the 11th most robustly induced gene in this subgroup [16].

The Guanylate-Binding Proteins are possibly the most abundantly induced genes upon IFN-γ stimulation [19]. Three of the members of this family are associated with breast cancer: GBP-1, GBP-2, and GBP-5 [14,16,20,21,22,23,24,25,26,27,28]. As described above, GBP-1 and GBP-5 were identified as prominent members of gene expression profiles correlated with improved prognosis [14,16].

GBP-1 is a part of gene signatures that correlate with improved breast cancer prognosis [13,14,16,21,29] (Table 1). It was also identified in a study of TNBC primary tumors using proteomics to identify proteins that correlate with length of time to recurrence [21]. Low expression of GBP-1 and STAT1 are associated with poorer RFS in these basal-like TNBCs [21]. GBP-1 also inhibits tumor cell growth by inhibiting angiogenesis [9,10,30]. In addition, it directly inhibits the growth of some epithelial tumor cell lines [30,31,32] and inhibits actin dynamics [33,34,35]. Most recently, GBP-1 was identified as a predictive marker for improved immunotherapy response in an analysis of multiple cancer types [36]. In this study, GBP-1 expression positively correlated with immune checkpoint genes and immune cell infiltration [36]. Tumors with elevated GBP-1 showed longer OS and greater clinical benefits from immunotherapy [36]. However, despite most of the evidence favoring a positive, if not protective, role for GBP-1 in breast cancer, there are some studies that suggest the contrary [20,37,38,39]. It is unclear what scientific conditions are responsible for these differences. The data sets showing the correlation of GBP-1 expression with good prognosis almost always have STAT1 in the set, indicating the presence of an IFN-γ-driven gene profile. These tumors would be expected to have activated T-, B-, and NK cells [13,14,16]. Since GBPs are some of the most robustly induced genes upon IFN-γ exposure, that also leads to the question of whether the presence of GBP-1 in these gene signatures is just the byproduct of an IFN-γ gene response or whether GBP-1 actually contributes to improved prognosis. As such, a further question is, if GBP-1 is a direct contributor, whether this activity cell is autonomous within the tumor cells themselves or the consequence of the infiltrating activated T-, B-, and NK cells, or a combination of both.

GBP-5 is the second of the three GBPs associated with breast cancer. Once again, however, there are some disparities in descriptions of the role of GBP-5 in breast cancers (Table 2). While GBP-5 has been associated with improved prognosis in breast cancers [16,26,28], one group of investigators published that GBP-5 correlated with good prognosis in TNBC samples [26] and then used a different set of array data to suggest it correlated with poor prognosis [27]. It is not immediately clear how to reconcile those findings. GBP-5 is elevated in a subset of basal-like TNBCs with significantly improved survival [29]. These tumors were also elevated in IFN-γ-induced chemokines [29]. In alignment with this thought, basal-like breast cancers can be divided into those with high versus low M1 polarization markers and high levels of M1 markers correlate with improved prognosis [29]. Those markers include GBP-5 [29].

Unlike GBP-1 and GBP-5, all the literature on GBP-2 and breast cancer indicates that it is protective [22,23,24,25,40,41,42] (Table 3). The literature on GBP-2 suggests that it would promote a good prognosis in breast cancer by inhibiting cell migration/invasion [22,23,40,41]. GBP-2 is down-regulated by promoter methylation in about 73.2% of breast cancers [25]. This inhibition of GBP-2 was found primarily in TNBCs, higher stages, and lymph node positive tumors [25]. However, as observed for GBP-1, GBP-2 has been suggested to enhance GBM cell invasion [43]. The role of GBP-2 in cancers may be either tumor type specific and/or EGF versus IFN-γ environment driven.

GBP-2 was first demonstrated to inhibit the ability of cells to make the rapid reorganization of the actin cytoskeleton required for cell spreading when prompted by integrin engagement or treatment with either PDGF or TNF-α [40,41]. This cytoskeletal inhibition was accompanied by the inhibition of Rac1. However, GBP-2 also inhibited both the basal and TNF-α induced expression of MMP-9 [41]. GBP-2 inhibits the activation of NF-κB by TNF-α [41]. Upon TNF-α treatment, IκBα is degraded and p65 goes to the nucleus but it does not bind to the NF-κB promoter element at the MMP-9 gene [41]. These changes could play a role in the inhibition of cell migration/invasion and metastasis.

GBP-2 was most recently studied in the 4T1 model of murine breast cancer [24]. This model was developed from a spontaneously arising breast cancer in a BALB/c mouse [44]. Multiple cell lines were isolated from this heterogenous breast tumor [44]. The 4T1 cell line is highly aggressive when injected into the mammary fat pats of BALB/c mice, while the 67NR cell line does not leave the primary site. [44]. 4T1 cells do not express GBP-2, while 67NR cells do [24]. Forced expression of GBP-2 in 4T1 cells inhibits their migration, while knockdown of GBP-2 in 67NR cells promotes their migration and the formation of invadosomes [24]. GBP-2 inhibits breast cancer cell migration by inhibiting the activation of Rac1, while promoting the activation of CDC42 and RhoA [24]. GBP-2 has also been suggested to inhibit breast cancer cell migration and invasion by inhibiting mitochondrial fission by blocking the ability of Drp1 to translocate to the mitochondria [23]. Inhibition of mitochondrial fission inhibits breast cancer cell invasion and metastasis [45].

One point that remains unclear is whether these three proteins are bystanders in breast cancers, as the consequence of the presence of IFNs, or whether they are actively involved in outcomes. We have determined whether each of these GBPs can serve as a single agent predictor of breast cancer recurrence free survival (RFS), overall survival (OS), or distance metastasis survival (DMFS) using a group of publicly available microarray databases. We have performed the analyses including all breast cancers but also after grouping the samples into ER+, ER+/HER2−, HER2+, ER−, and TNBC subtypes. We found that the predicted behavior of individual GBPs varied by breast cancer hormone status, based on these subgroupings. We also correlated these results with published studies on these GBPs in breast cancers, as presented further in this paper.

## 2. Materials and Methods

### Gene Expression Profiling and Data Processing

To address the potential relevance of GBP-1 as an independent indicator of breast cancer prognosis, Kaplan–Meier plots were generated to predict the outcome of low versus high GBP-1 expression on Recurrence-Free Survival (RFS), Overall Survival (OS), and Distant Metastasis-Free Survival (DMFS) for different breast cancer subtypes The program Km plot [46,47] was used to analyze the data from the following publicly available microarray data sets: E-MTAB-365 (*n* = 537), E-TABM-43 (*n* = 37), GSE11121 (*n* = 200), GSE12093 (*n* = 136), GSE12276 (*n* = 204), GSE1456 (*n* = 159), GSE16391 (*n* = 55), GSE16446 (*n* = 120), GSE16716 (*n* = 47), GSE177705 (*n* = 196), GSE17907 (*n* = 54), GSE18728 (*n* = 61), GSE19615 (*n* = 115), GSE20194 (*n* = 45), GSE20271 (*n* = 96), GSE2034 (*n* = 286), GSE20685 (*n* = 327), GSE20711 (*n* = 90), GSE21653 (*n* = 240), GSE22093 (*n* = 68), GSE25066 (*n* = 507), GSE2603 (*n* = 99), GSE26971 (*n* = 276), GSE29044 (*n* = 79), GSE2990 (*n* = 102), GSE31448 (*n* = 71), GSE31519 (*n* = 67), GSE32646 (*n* = 115), GSE3494 (*n* = 251), GSE36771 (*n* = 107), GSE37946 (*n* = 41), GSE41998 (*n* = 279), GSE42568 (*n* = 121), GSE43358 (*n* = 57), GSE43365 (*n* = 111), GSE45255 (*n* = 139), GSE4611 (*n* = 153), GSE46184 (*n* = 74), GSA48390 (*n* = 81), GSE50948 (*n* = 156), GSE5327 (*n* = 58), GSE58812 (*n* = 107), GSE61304 (*n* = 62), GSE65194 (*n* = 164), GSE6532 (*n* = 82), GSE69031 (*n* = 130), GSE7390 (*n* = 198), GSE76275 (*n* = 265), GSE78958 (*n* = 424), and GSE9195 (*n* = 77). All of the databases used the HG-U133A Affymetrix microarray platform. Km plot normalizes the raw CEL files using MAS5 within the R environment (www.r-project.org (accessed on 31 December 2021) and uses the affy Bioconductor library [46]. For gene array analyses, there was no filtering for intrinsic subtype (histology), grade, stage, lymph node status, race, or treatment. All breast cancers included all tumors regardless of hormone status. ER+ data represents only those breast cancers positive for estrogen receptor but with any other hormone status. ER+/HER2− contains the data from all ER+ breast tumors than were also HER2−. These could be positive or negative for progesterone (PR). HER2+ tumors included those that were positive for HER2+ and had any other hormone receptor status. ER− tumors are those without amplified ER but any other hormone receptor status. For gene array analysis of TNBC tumors, only the ER negative, PR negative, and unamplified HER2 tumors were analyzed. The patients were split by the median value of GBP expression into low versus high expression. The data in the tables is presented as Hazard Ratio (HR) immediately followed by the calculated HR. The range of numbers within the paratheses is the 95% confidence interval. P represents the LogRank P. Bold values are those that indicate that higher expression of the GBP significantly correlates with improved prognosis. Data that are both bolded and in italics describe a situation where elevated GBP-1 is significantly correlated with poorer prognosis.

## 3. Results

### 3.1. GBP-1

**Figure 1:**
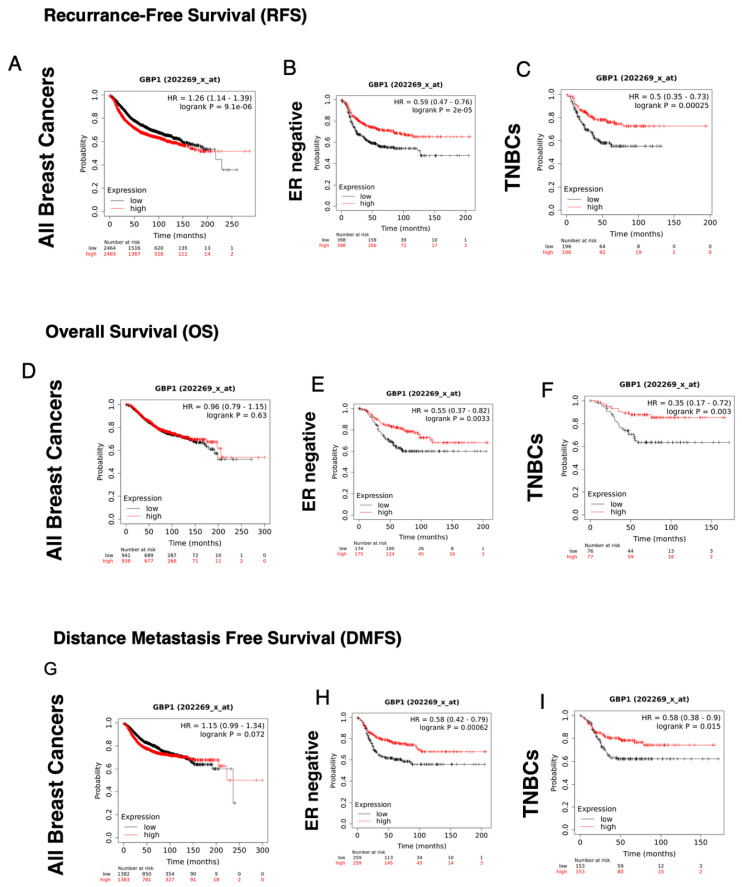
High levels of GBP-1 correlate with better recurrence-free (RFS), overall survival (OS), and distance metastasis-free survival (DMFS) in human ER− and TNBC breast cancers. Km plots were performed for RFS for those tumors with high versus low levels of GBP-1 expression for (**A**) all breast cancers, (**B**) ER− breast cancers, and (**C**) TNBCs. Km plots were performed for OS of tumors with high versus low GBP-1 expression for (**D**) all breast cancers, (**E**) ER− breast cancers, and (**F**) TNBCs. Km plots were performed for DMFS for those tumors with high versus low GBP-1 expression for (**G)** all breast cancers, (**H**) ER− breast cancers, and (**I**) TNBCs.

If GBP-1 directly participates in breast cancer progression and/or prognosis as a single gene product, it would be expected to be prognostic as an independent indicator. To address the potential relevance of GBP-1 as an independent indicator of breast cancer prognosis, Kaplan–Meier plots were generated to predict the outcome of low versus high GBP-1 expression on Recurrence-Free Survival (RFS), Overall Survival (OS), and Distant Metastasis-Free Survival (DMFS) for different breast cancer subtypes (Table 4). GBP-1 correlates with significantly poorer RFS when examined as a single gene for a cohort of breast cancer patients that contains all forms of breast cancers (Figure 1A, Table 4). When analyzing all breast cancers, GBP-1 also has no effect as a single gene on OS or DMFS (Figure 1D,G, Table 4). As a single gene, GBP-1 also does not contribute to RFS, OS, or DMSF in ER+, ER+/HER2−, or HER2+ breast tumors (Table 4). In breast cancers of these genetic backgrounds, GBP-1 expression is not sufficient to independently predict the outcome.

Alternatively, GBP-1 correlates with significantly improved RFS, OS, and DMFS in both TNBC and ER− breast cancers (Figure 1, Table 4). Common to both types of tumors is the lack of elevated ER. The Km data suggest that in ER− tumors GBP-1 behaves differently than in tumors with elevated ER (Table 4).

### 3.2. GBP-5

**Figure 2:**
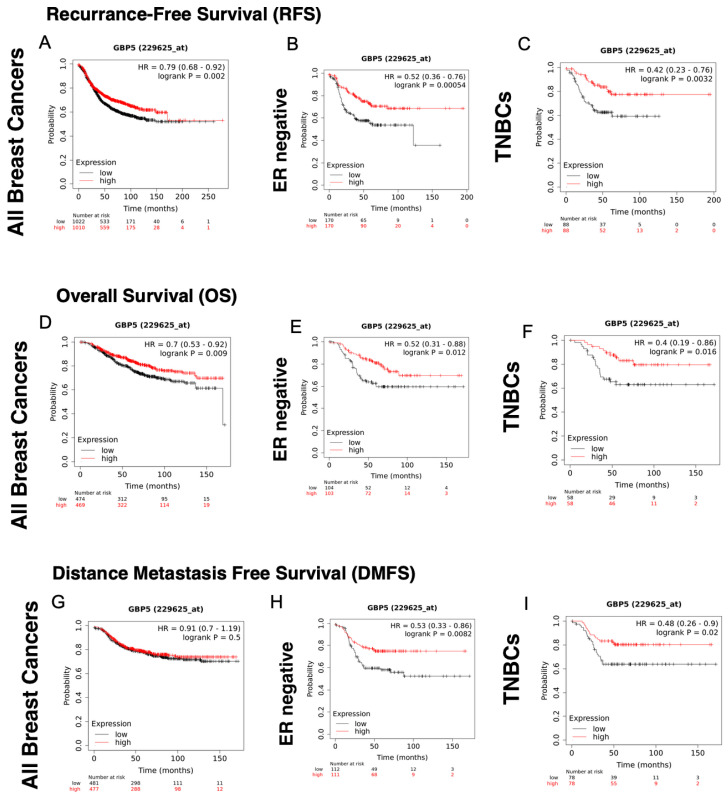
GBP-5 correlates with better recurrence-free (RFS) and overall survival (OS) in all breast cancers. The probability of RFS versus time for breast cancers of all types, stages, and grades was plotted for those tumors with high and low levels of GBP-5 expression (**A**). The probability of RFS versus time was plotted for ER−, PR−, and HER2− (TNBC) breast cancers for high versus low levels of GBP-5 expression (**B**,**C**). The OS of patients of patients with all subtypes, stages, and grades was plotted for those tumors with high versus low GBP-5 expression versus time (**D**). The probability of OS versus time was plotted for ER−, PR−, and HER2− (TNBC) breast cancers for high versus low levels of GBP-5 expression (**E**,**F**). The DMFS of patients of patients with all subtypes, stages, and grades was plotted for those tumors with high versus low GBP-5 expression versus time (**G**). The probability of DMFS versus time was plotted for ER−, PR−, and HER2− (TNBC) breast cancers for high versus low levels of GBP-5 expression (**H**,**I**).

Unlike GBP-1, GBP-5 correlates with improved RFS and OS as a single gene in a cohort containing all breast cancers (Figure 2, Table 5). Similar to GBP-1, GBP-5 correlates with improved RFS and OS in ER− and TNBC (Figure 2 and Table 5). While RFS in HER2+ tumors is improved with the expression of GBP-5, the results for OS are probe set–specific and therefore not consistent (Table 5). Like GBP-1, GBP-5 is not a good prognostic indicator in ER+ breast cancers. Whether GBP-5 correlates with improved DMFS in TNBC remains unclear due to probe set discrepancies, although it appears to do so for ER− breast cancers (Table 5).

Because the patterns of expression of GBP-5 do not show the same outcomes as those of GBP-1, we predict that their activities are not the same. However, very little is known about the function of GBP-5. At this point it cannot be ruled out that whether GBP-5 is present in an IFN-γ environment or in an EGFR environment may make a big difference in its functions. The recent publication of a paper suggesting GBP-5 drives glioblastoma malignancy suggests that cell type and cellular environment will be important [48].

### 3.3. GBP-2

**Figure 3:**
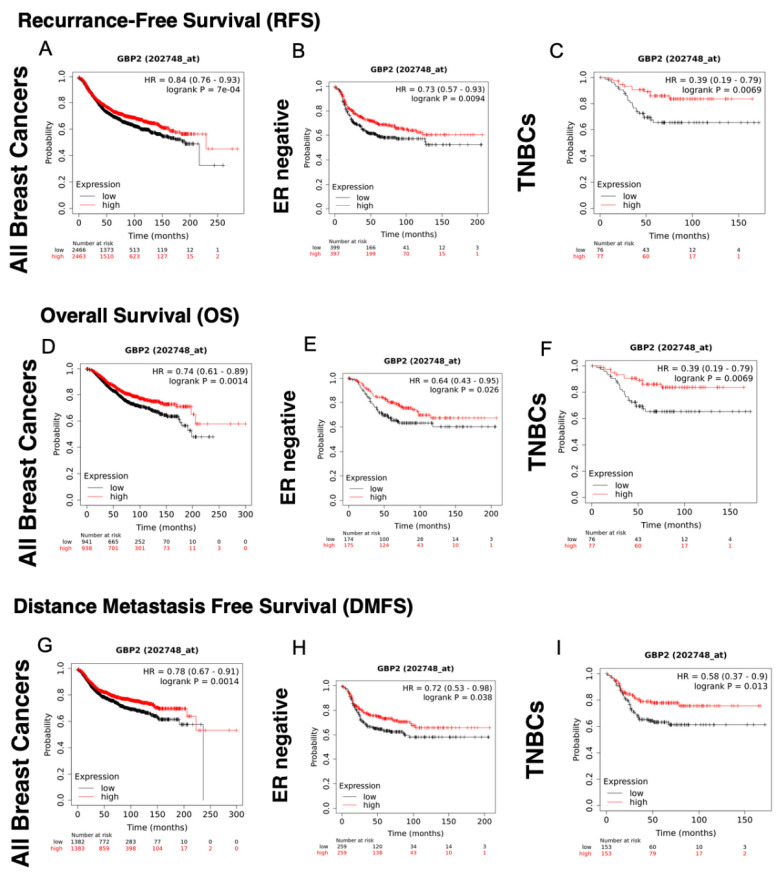
GBP-2 correlates with better recurrence-free (RFS), overall survival (OS), and Distance Metastasis-free Survival (DMFS) in a cohort containing all breast cancers. The probability of RFS versus time for breast cancers of all types, stages, and grades was plotted for those tumors with high and low levels of GBP-2 expression (**A**). The probability of RFS versus time was plotted for ER−, PR−, and HER2− (TNBC) breast cancers for high versus low levels of GBP-2 expression (**B**,**C**). The OS of patients of patients with all subtypes, stages, and grades was plotted for those tumors with high versus low GBP-2 expression versus time (**D**). The probability of OS versus time was plotted for ER−, PR−, and HER2− (TNBC) breast cancers for high versus low levels of GBP-2 expression (**E**,**F**). The DMFS of patients of patients with all subtypes, stages, and grades was plotted for those tumors with high versus low GBP-2 expression versus time (**G**). The probability of DMFS versus time was plotted for ER−, PR−, and HER2− (TNBC) breast cancers for high versus low levels of GBP-2 expression (**H**,**I**).

GBP-2 correlated with improved RFS in the cohort containing all breast cancers and in ER− and TNBC (Table 6). The same was observed for OS (Figure 3, Table 6). GBP-2 appeared to be protective on a larger scale when DMFS was analyzed. In addition to correlating with improved DMFS in the cohort of all breast cancers, TNBCs, and ER− breast cancers, it correlated with improved DMFS in ER+/HER2− tumors (Table 6). These data are consistent with GBP-2 inhibiting breast cancer cell migration/invasion [23,24].

## 4. Discussion

### 4.1. GBP-1

The predictive value of GBP-1 as a single agent in breast cancer appears to only be applicable to ER− and TNBC breast cancers (Figure 1, Table 4). This is despite literature that suggests it has general protective properties. What is becoming increasingly clear is that GBP-1′s activity is dependent on tumor type and tumor environment [20,30,31,32,36,38,49,50,51,52,53,54,55,56,57,58,59,60,61,62,63,64,65,66,67,68,69]. GBP-1 correlates with poor prognosis and increased invasion/metastasis in a variety of tumors with a growth factor–driven gene signature, particularly glioblastoma [52,55,58,59]. In glioblastoma, EGFR signaling induces GBP-1, which promotes cell invasion in vitro and in vivo and cell proliferation in vivo [52,55,58,59]. GBP-1, when induced by EGFR signaling in GBM cells, upregulates matrix metalloproteinase-1 (MMP-1) [59]. This upregulation by EGFR signaling is GBP-1-dependent and contributes to glioblastoma invasion [59]. IFN-γ treatment of glioblastoma cells to induce GBP-1 does not induce MMP-1 [59]. These data suggest that GBP-1 behaves differently in different types of breast cancers, based on differences in their genetic profiles and/or their surrounding environment. GBP-1 behaves more favorably in an environment driven by an IFN-γ immune response [14,16]. This has also been observed for GBP-1 in colon cancer [57,70]. The recent finding that GBP-1 can be unregulated in breast cancer cell lines by epidermal growth factor receptor (EGFR) signaling might provide clues on why GBP-1 is not protective when all types of breast cancers are considered as a group (Figure 1) [20]. GBP-1 is also downstream of EGFR signaling in glioblastomas [55,58,59,71]. While treatment of GBM cells with either EGF or IFN-γ induces the expression of GBP-1, only EGF treatment induces the expression of matrix metalloproteinase 1 (MMP1) and promotes tumor cell migration/invasion [59]. GBP-1 promotes GBM invasion and the EGF induction of MMP1 is GBP-1-dependent [59]. If GBP-1 behaves differently in a growth factor–driven environment than an IFN-γ-driven environment, this also would be consistent with GBP-1 not being a independent predictor of better prognosis, and why it needs STAT1 to be present to distinguish between a growth factor versus an IFN-γ-driven environment.

One way that GBP-1 could promote a better prognosis in breast cancers is through its ability to inhibit angiogenesis [9,10,30,70]. IFN-γ induces GBP-1 in endothelial cells cultured in vascular endothelial growth factor (VEGF) and beta-fibroblast growth factor (bFGF) to promote proliferation, and GBP-1 inhibits their proliferation, migration, invasion, and ability to form tubular structures in vitro [9,10]. GBP-1 is also inversely correlated with endothelial cell proliferation in Kaposi sarcomas and inflammatory cytokine induced inflammation in vivo [9,72]. GBP-1 inhibits endothelial cell spreading and migration, in part by inducing the expression of integrin alpha 4 [35]. IFN-γ induction of GBP-1 in endothelial cells inhibits the expression of MMP-1, which results in decreased endothelial invasion [10]. In addition, purified GBP-1 inhibits actin polymerization in vitro [34], which should slow migration/invasion.

Where GBP-1 is predictive of improved prognosis in breast cancers is when it is part of an IFN-γ gene signature [13,14,15]. In TNBCs, these are the basal-like immune activated tumors [16]. The observation that GBP-1 promoted improved RFS, OS, and DMSF in TNBCs is consistent with the data on basal-like immune activated (BLIA) TNBCs, where elevated GBP-1 and GBP-5 correlate with improved prognosis [16]. That subclassification of basal-like TNBCs was associated with elevation of gene signatures associated with T-, B-, and NK cell activation [16]. Elevated GBP-1 was also observed in a subset of basal-like TNBCs with elevated M1 macrophages [29]. These tumors also showed elevated levels of IFN-γ-induced chemokines and improved prognosis [29].

### 4.2. GBP-5

While GBP-5 correlates with improved prognosis in TNBCs, it is unclear why (Figure 2, Table 5). So far, most of what is known about GBP-5 in cancers is strictly correlative. The correlation with DMFS suggests that GBP-5 inhibits breast cancer metastasis but there is no data on GBP-5 function to confirm that. GBP-5 promotes NLRP3 inflammasome assembly, but it is unclear how that influences breast cancer prognosis [73].

Unlike other GBPs, GBP-5 has three mRNA splice variants that encode two different proteins [74]. The two proteins are designated as GBP-5a/b, which is full length, and GBP-5ta, which is missing 97 amino acids from the C-terminus [74]. Consequently, GBP-5ta is missing its CaaX sequence, which could result in dysregulated membrane targeting. Unlike GBP-1, GBP-5a/b and GBP-5ta hydrolyze GTP to only GDP and do not produce or bind to GMP [75]. While both isoforms are identified by RT-PCR, GBP-5ta protein was only found in monocytes and at very low levels [74]. However, screening of cutaneous T-cell lymphoma (CTCL) tumors found only GBP-5ta in seven or seven tumors. Both isoforms were expressed in four out of four CTCL cell lines. While eight of nine melanoma cell lines expressed GBP-5a/b, four of the nine also had low levels of GBP-5ta. This led to the calling GBP-5ta a tumor cell specific splice variant [74]. The biochemical properties of GBP-5a/b and GBP-5ta are not very different [75].

### 4.3. GBP-2

After its cloning, GBP-2 was shown to inhibit cell spreading, in part by inhibiting the activation of Rac1 [40]. Significantly, this inhibition was observed after integrin stimulation, PDGF and/or TNF-α treatment [40,41]. Work in breast cancer cells extended this work by showing that GBP-2 inhibits cell migration and invadosome formation, accompanied by inhibition of Rac1 and activation of Cdc42 and RhoA [24]. This is consistent with the improved DMSF with high GBP-2 in all subtypes of breast cancer, except HER2+ (Table 6). Also consistent is the role of GBP-2 in inhibiting mitochondrial fission, which inhibits breast cancer cell migration/invasion.

GBP-2 also inhibits the TNF-α induction of matrix metalloproteinase-9 (MMP-9) [41], known to play a role in breast cancer invasion. In the presence of GBP-2, TNF-α induces the degradation of IκBα and p65 is released and translocates into the nucleus, but p65 does not bind to the NF-κB site in the promoter of the MMP-9 gene [41]. This inhibition of p65 binding only seems to affect a subset of NF-κB promoter elements and may selectively regulate the expression of TNF-α induced cytokines [41]. Clearly, more research on how GBP-2 promotes a better prognosis in breast cancer is needed.

GBPs are members of the dynamin superfamily of large GTPases [76,77]. As such, they can form dimers and higher order complexes [76,77,78]. In vitro, they can be driven to form homodimers by the binding of GTP analogues [76,77,78]. GBP-1, GBP-2, and GBP-5 are isoprenylated and this lipid modification is required for membrane recruitment [79]. Recruitment of the members of the family to specific intracellular membranes occurs in a hierarchical fashion [79]. Studies with transfected cells showed that GBP-1 could relocalize GBP-5 and GBP-2. GBP-5 could relocalize GBP-2 and the prenylated GBPs could subsequently recruit the unprenylated family members [79,80]. This suggests that heterodimerization or higher order structures can modulate membrane association of the family members. It is unclear how/if heterodimerization influences GBP function.

## 5. Conclusions

While our understanding of GBP-1, -2, and -5 in breast cancers is improving, much of what we know is still correlative. GBP-5 is correlated with improved prognosis in TNBCs [16] and ER− breast cancers (Table 5) but the molecular activities of GBP-5 that contribute to this are unknown. Of the GBPs involved in breast cancer, the least is known about the function of GBP-5. GBP-1 expression in cancers can be a double-edged sword (Table 4). Within the context of an IFN-γ-driven gene signature, GBP-1 promotes a better prognosis in breast and colon cancer [13,14,57,70]. However, in a variety of other cancers with growth factor–driven gene signatures, GBP-1 promotes cell motility and poor prognosis [20,32,33,38,40,42,44,46,47,54,55,56,57,58,59,60,61,62,63,64,65,66,67,68,69,70,71]. Since some breast cancers are growth factor–driven, this may explain why GBP-1 is only correlated with improved prognosis in a subset of breast cancers without receptor amplification. It is increasingly clear that GBP-1 cannot be used as a single marker for tumor prognosis but needs to be considered within the tumor type and growth factor environment. This is despite the fact that GBP-1 can inhibit tumor angiogenesis, inhibit breast cancer proliferation, and directly inhibit actin polymerization [9,10,30,34]. GBP-2 also promotes a better prognosis in breast and colon cancer [23,24,81] (Table 6), but promotes glioblastoma progression [43,82].

## Figures and Tables

**Table 1:** Manuscripts addressing GBP-1 in breast cancer.

Cell Lines	Clinical Samples	Results	Reference
-	Breast cancer samples	GBP-1 and STAT1 are part of a 5 gene signature that correlates with improved RFS in all breast cancers. GBP-1 expression is highest in infiltrating cells but was also expressed in the tumor cells of recurrence-free patients.	Ascierto, 2013 [13]
-	TNBC tumor samples	Subtyped TNBCs into 4 subgroups. Two subgroups were of basal histology. Basal-like Immune Activated (BLIA) tumors had elevated expression for genes for T-cell, B-cell, and NK activation. Further, IFN-γ stimulated genes. GBP-5 was the 3rd–5th most robustly induced gene in BLIA tumors, and GBP-1 was the 11th. BLIA tumors are predicted to have greater than 85% RFS over 10 years, much better than other forms of TNBC.	Burstein, 2015 [16]
TS/A	-	Forced expression of GBP-1 in TS/A cells resulted in smaller tumors in immune competent mice. Not accompanied by decrease in infiltrating cells. Reduced Ki67. Reduced level of VEGF-A both in vitro and in vivo.	Lipnik, 2010 [30]
MCF-7, SKBR3, MDA-MB-468, MDA-MB-436, MDA-MB-231, BT549, T47D	Breast cancer samples	Upregulated genes in TNBCs were analyzed for druggability using ChRMBL Studel. GBP-1 was ranked second in the upregulated genes based on druggability. KD of GBP-1 inhibited proliferation in a subgroup of TNBC cell lines. EGFR can drive GBP-1 expression in breast cancer.	Quintero, 2017 [20]
SAS, HepG2, KB, MM102 cells	-	Cells made clinically cells radioresistant (CRR). All CRR cells expressed elevated GBP compared to parental cells. KD of GBP reduced radioresistance.	Fukumoto, 2014 [37]
MDA-MB-231, MDA-MB-231-BM, SUM159PT	Human primary T-cells	Co-culturing activated T-cells with breast cancer cells increased their crossing of artificial blood brain barrier (BBB). GBP-1 was upregulated in the tumor cells after incubation with activated T-cells. KD of GBP-1 in tumor cells reduced crossing of the BBB after incubation with activated T-cells.	Mustafa, 2018 [38]
Jurkat cells	Primary human T-cells	Silencing of GBP-1 increases T-cell spreading and surface expression of TCR/CD3 and CD45. Modulates early TCR signaling.	Forster, 2014 [33]

**Table 2:** Manuscripts addressing GBP-5 and breast cancer. If you want to move Table 1 to the introduction then you should relabel this Table 2 and move it there also.

Cell Lines	Clinical Samples	Results	Reference
-	TNBCs	GBP-5 is 5th most robustly induced gene in BLIA TNBC tumors with gene signatures of IFN-γ, B-cell, T-cell, and NK cell activation. Significantly improved survival compared to other TNBCs, particularly other basal TNBCs.	Burstein, 2015 [16]
MDA-MB-231, Hs578T	TNBCs	High expression of GBP-5 correlated with improved RFS and PRS in TNBCs. GBP-5 not expressed in normal breast epithelial cells but expressed in 5/7 TNBC cell lines. Contributes to paclitaxel sensitivity. Suggest GBP-5 promotes TNBC protection by activating Akt/mTOR and inhibiting autophagy.	Cheng, 2021 [26]
MDA-MB-231, Hs578T	TNBCs	GBP-5 expression correlates with poor prognosis in TNBCs. KD of GBP-5 inhibited cell migration and activity from both GAS and NF-kB promoter elements.	Cheng, 2021 [27]
-	Breast Cancer Samples	Mutations in tumors with high PD1 and PD-L1 were associated with GBP-5 expression and good prognosis. Also associated with immune infiltration of the tumors.	Cimas, 2020 [28]

**Table 3:** Manuscripts addressing GBP-2 and breast cancer. However, it should be noted that the Godoy et al. manuscript listed the incorrect Affymetrix probe set for their analysis of GBP-2 in breast cancer [22]. The probe set they listed was for an Ig light chain subunit.

Cell Lines	Clinical Samples	Results	Reference
766 node negative breast cancers	-	Elevated GBP-2 correlated with longer time to distant metastasis in highly proliferating ER+ tumors with infiltrating T-cells (as judged by gene signature).	Godoy, 2014 [22]
MDA-MB-231 and MDA-MB-436	-	GBP-2 interacts with Drp1 to both inhibit mitochondrial fission and cell migration.	Zhang, 2017 [23]
4T1, 67NR murine breast cancer cells	-	GBP-2 inhibits cell migration by inhibiting Rac1 activation and activating CDC42 and RhoA. Inhibits invadosome formation.	Nyabuto, 2021 [24]
NIH 3T3 fibroblasts, B16 melanoma	-	GBP-2 inhibits cell spreading downstream of integrins, PDGF, and TNF-α treatment. Inhibits activation of Rac1 and PI3-K when cells plated on fibronectin.	Messmer-Blust, 2010 [40]
NIH3T3 cells	-	GBP-2 inhibits TNF-α induction of matrix metalloprotease-9 (MMP-9) by inhibiting the binding of NF-κB p65 to the MMP-9 promoter.	Balasubramanian, 2011 [41]
TE-1 squamous cell carcinoma cells	-	GBP-2 is a p53 responsive gene.	Guimaraes, 2009 [42]
-	Breast cancer and normal breast tissue, plasma	Levels of GBP-2 are reduced in breast tumors compared to normal breast tissue. GBP-2 is reduced in TNBC tumors, higher stages of breast cancers, and in node positive tumors compared to other breast tumors. The GBP-2 promoter in about 87% of breast cancers was methylated. GBP-2 was downregulated in 73% of breast cancers, 26% had normal GBP-2 expression, and none showed elevation of GBP-2. The methylation status of the GBP-2 promoter in tumors matched with the methylation status of cell free DNA isolated from the plasma. GBP-2 promoter was methylated in 100% of stage III or IV breast tumors.	Rahvar, 2020 [25]

**Table 4:** Data from all three GBP-1 probe sets of the HG-U133A Affymetrix microarray were analyzed for Recurrence-Free Survival (RFS), Overall Survival (OS), and Distant Metastasis-Free Survival (DMSF) using KmPlotter. The data are presented as Hazard Ratio (HR) immediately followed by the HR number. The range of numbers within the paratheses is the 95% confidence interval. *p* represents the LogRank *p*. Bold values are those that indicate that higher expression of GBP-1 significantly improves that outcome. Data that are both in bold font and in italics describe a situation where elevated GBP-1 is significantly correlated with poorer RFS.

GBP-1	Affymetrix Probe Sets		
	202269_x_at	231577_s_at	202270_at
**Recurrence-Free Survival**			
All Breast Cancers	***HR = 1.26 (1.14–1.39),*** ***p = 9.1 × 10^−6^***	***HR = 1.21 (1.04–1.41),*** ***p = 0.012***	***HR = 1.19 (1.07–1.31),*** ***p = 0.00088***
ER+	HR = 1.02 (0.87–1.19), *p* = 0.83	HR = 1.18 (0.89–1.58), *p* = 0.25	HR = 1 (0.86–1.17), *p* = 0.97
ER+/HER2−	HR = 1.02 (0.86–1.2), *p* = 0.85	HR = 1.1 (0.8–1.51), *p* = 0.55	HR = 1.1 (0.93–1.3), *p* = 0.28
HER2+	HR = 0.87 (0.7–1.08), *p* = 0.19	HR = 1 (0.74–1.35), *p* = 1	**HR = 0.69 (0.55–0.86),** ***p* = 00081**
ER−	**HR = 0.59 (0.47–0.76),** ***p* = 2 × 10^−5^**	**HR = 0.58 (0.4–0.85),** ***p* = 0.0042**	**HR = 0.58 (0.46–0.74),** ***p* = 1 × 10^−5^**
TNBC	**HR = 0.5 (0.35–0.73),** ***p* = 0.00025**	**HR = 0.31 (0.17–0.59),** ***p* = 0.00016**	**HR = 0.45 (0.31–0.66),** ***p* = 2.1 × 10^−5^**
**Overall Survival**			
All Breast Cancers	HR = 0.96 (0.79–1.15), *p* = 0.63	HR = 0.91 (0.7–119), *p* = 0.5	HR = 1.03 (0.85–1.24), *p* = 0.75
ER+	HR = 1 (0.72–1.38), *p* = 0.99	HR = 0.94 (0.46–1.96), *p* = 0.88	HR = 1.08 (0.78–1.49), *p* = 0.64
ER+/HER2−	HR = 1.05 (0.73–1.5), *p* = 0.81	HR = 0.92 (0.39–2.17), *p* = 0.85	**HR = 0.66 (0.45–0.95),** ***p* = 0.023**
HER2+	HR = 0.7 (0.49–1.01), *p* = 0.58	HR = 0.9 (0.55–1.49), *p* = 0.69	HR = 1.12 (0.78–1.62), *p* = 0.53
ER−	**HR = 0.55 (0.37–0.82),** ***p* = 0.0033**	**HR = 0.59 (0.35–0.98),** ***p* = 0.039**	**HR = 0.55 (0.37–0.82),** ***p* = 0.003**
TNBC	**HR = 0.35 (0.17–0.72),** ***p* = 0.003**	**HR = 0.36 (0.16–0.78),** ***p* = 0.0071**	**HR = 0.49 (0.25–0.98),** ***p* = 0.039**
**Distant Metastasis-Free Survival**			
All Breast Cancers	HR = 1.15 (0.99–1.34), *p* = 0.072	HR = 1.07 (0.82–1.39), *p* = 0.63	HR = 1.16 (1–1.36), *p* = 0.053
ER+	HR = 1.13 (0.86–1.49), *p* = 0.37	HR = 2.14 (0.98–4.65), *p* = 0.05	HR = 0.99 (0.76–1.31), *p* = 0.96
ER+/HER2−	HR = 1.09 (0.81–1.47), *p* = 0.58	HR = 1.91 (0.69–5.29), *p* = 0.2	HR = 1 (0.74–1.35), *p* = 0.99
HER2+	**HR = 0.64 (0.45–0.89),** ***p* = 0.0083**	HR = 0.86 (0.54–1.35), *p* = 0.51	HR = 0.73 (0.53–1.03), *p* = 0.07
ER−	**HR = 0.58 (0.42–0.79),** ***p* = 0.00062**	**HR = 0.61 (0.38–0.98),** ***p* = 0.04**	**HR = 0.58 (0.42–0.79),** ***p* = 0.00059**
TNBC	**HR = 0.58 (0.38–0.9),** ***p* = 0.015**	**HR = 0.47 (0.25–0.88),** ***p* = 0.017**	**HR = 0.52 (0.34–0.81),** ***p* = 0.003**

**Table 5:** Data from both GBP-5 probe sets of the HG-U133A Affymetrix microarray were analyzed for Recurrence-Free Survival (RFS), Overall Survival (OS), and Distant Metastasis-Free Survival (DMSF) using KmPlotter. All breast cancers included breast cancers of all histologies, hormone status, and grade. ER+ represents only those breast cancers positive for estrogen receptor but with any other hormone status. ER+/HER2− contains the data from all ER+ breast tumors than were also HER2−. There could be positive or negative for progesterone (PR). HER2+ tumors included those that were positive for HER2+ and had any other hormone receptor status. The data are presented as Hazard Ratio (HR) immediately followed by the HR. The range of numbers within the paratheses is the 95% confidence interval. *p* represents the LogRank *p*. Bold values are those that indicate that higher expression of GBP-5 significantly improves that prognosis.

GBP-5	Affymetrix Probe Sets	
	229625_at	23581_at
**Recurrence-Free Survival**		
All Breast Cancers	**HR = 0.79 (0.68–0.92), *p* = 0.002**	**HR = 0.78 (0.67–0.91), *p* = 0.0015**
ER+	HR = 1.04 (0.78–1.38), *p* = 0.81	HR = 1.05 (0.79–1.39), *p* = 0.76
ER+/HER2−	HR = 1.1 (0.8–1.51), *p* = 0.55	HR = 1.02 (0.75–1.4), *p* = 0.9
HER2+	**HR = 0.58 (0.43–0.8), *p* = 0.00054**	**HR = 0.59 (0.43–0.8), *p* = 0.00057**
ER−	**HR = 0.56 (0.39–0.82), *p* = 0.0087**	**HR = 0.56 (0.39–0.82), *p* = 0.0088**
TNBC	**HR = 0.42 (0.23–0.76), *p* = 0.0032**	**HR = 0.46 (0.26–0.83), *p* = 0.0088**
**Overall Survival**		
All Breast Cancers	**HR = 0.7 (0.53–0.92), *p* = 0.009**	**HR = 0.72 (0.55–0.94), *p* = 0.017**
ER+	HR = 0.59 (0.28–1.25), *p* = 0.17	HR = 0.8 (0.39–1.67), *p* = 0.55
ER+/HER2−	HR = 0.63 (0.26–1.49), *p* = 0.28	HR = 0.72 (0.3–1.72), *p* = 0.46
HER2+	HR = 0.68 (0.41–1.13), *p* = 0.14	**HR = 0.6 (0.36–0.99), *p* = 0.045**
ER−	**HR = 0.52 (0.31–0.88), *p* = 0.012**	**HR = 0.4 (0.23–0.68), *p* = 0.00043**
TNBC	**HR = 0.4 (0.19–0.86), *p* = 0.016**	**HR = 0.41 (0.19–0.87), *p* = 0.017**
**Distant Metastasis-Free Survival**		
All Breast Cancers	HR = 0.91 (0.7–1.19), *p* = 0.5	HR = 0.96 (0.73–1.24), *p* = 0.74
ER+	HR = 1.32 (0.62–2.79), *p* = 0.47	HR = 1.37 (0.65–2.9), *p* = 0.41
ER+/HER2−	HR = 1.17 (0.43–3.15), *p* = 0.76	HR = 1.06 (0.4–2.82), *p* = 0.91
HER2+	HR = 0.74 (0.47–1.17), *p* = 0.2	HR = 0.74 (0.47–1.17), *p* = 0.19
ER−	**HR = 0.53 (0.33–0.86), *p* = 0.0082**	**HR = 0.61 (0.38–0.98), *p* = 0.09**
TNBC	**HR = 0.48 (0.26–0.9), *p* = 0.02**	HR = 0.61 (0.33–1.13), *p* = 0.11

**Table 6:** Data from both GBP-2 probe sets of the HG-U133A Affymetrix microarray were analyzed for Recurrence-Free Survival (RFS), Overall Survival (OS), and Distant Metastasis-Free Survival (DMSF) using KmPlotter. All breast cancers included breast cancers of all histologies, hormone status, and grade. ER+ represents only those breast cancers positive for estrogen receptor but with any other hormone status. ER+/HER2− contains the data from all ER+ breast tumors than were also HER2−. There could be positive or negative for progesterone (PR). HER2+ tumors included those that were positive for HER2+ and had any other hormone receptor status. The data are presented as Hazard Ratio (HR) immediately followed by the HR. The range of numbers within the paratheses is the 95% confidence interval. *p* represents the LogRank *p*. Bold values are those that indicate that higher expression of GBP-2 significantly improves that prognosis.

GBP-2	Affymetrix Probe Sets	
	202748_at	242907_at
**Recurrence-Free Survival**		
All Breast Cancers	**HR = 0.84 (0.76–0.93), *p* = 7 × 10^−4^**	**HR = 0.72 (0.62–0.84), *p* = 2.8 × 10^−5^**
ER+	HR = 0.86 (0.74–1.01), *p* = 0.061	HR = 0.97 (0.72–1.29), *p* = 0.81
ER+/HER2−	**HR = 0.83 (0.7–0.98), *p* = 0.031**	HR = 0.88 (0.65–1.21), *p* = 0.45
HER2+	HR = 0.77 (0.62–0.96), *p* = 0.022	HR = 0.85 (0.63–1.15), *p* = 0.3
ER−	**HR = 0.73 (0.57–0.93), *p* = 0.0094**	**HR = 0.73 (0.57–0.93), *p* = 0.0095**
TNBC	**HR = 0.59 (0.41–0.86), *p* = 0.0048**	**HR = 0.34 (0.18–0.64), *p* = 0.00045**
**Overall Survival**		
All Breast Cancers	HR = 0.74 (0.61–0.898), *p* = 0.0014	**HR = 0.6 (0.46–0.79), *p* = 0.00019**
ER+	HR = 0.75 (0.55–1.04), *p* = 0.085	HR = 0.49 (0.23–1.05), *p* = 0.061
ER+/HER2−	HR = 0.72 (0.5–1.03), *p* = 0.071	HR = 0.44 (0.18–1.09). *p* = 0.068
HER2+	HR = 0.82 (0.57–1.17), *p* = 0.27	HR = 0.89 (0.54–1.47), *p* = 0.64
ER−	HR = 0.64 (0.43–0.95), *p* = 0.026	**HR = 0.64 (0.43–0.95), *p* = 0.027**
TNBC	HR = 0.39 (0.19–0.79), *p* = 0.0069	**HR = 0.34 (0.16–0.75), *p* = 0.0052**
**Distant Metastasis-Free Survival**		
All Breast Cancers	**HR = 0.78 (0.67–0.91), *p* = 0.0014**	HR = 0.81 (0.62–1.06), *p* = 0.13
ER+	**HR = 0.65 (0.49–0.86), *p* = 0.0022**	HR = 0.84 (0.39–1.78), *p* = 0.64
ER+/HER2−	**HR = 0.66 (0.49–0.9), *p* = 0.0079**	**HR = 0.66 (0.49–0.9), *p* = 0.0079**
HER2+	HR = 0.73 (0.52–1.02), *p* = 0.065	HR = 0.9 (0.57–1.42), *p* = 0.65
ER−	**HR = 0.72 (0.53–0.98), *p* = 0.038**	HR = 0.75 (0.47–1.2), *p* = 0.23
TNBC	**HR = 0.58 (0.37–0.9), *p* = 0.013**	**HR = 0.42 (0.22–0.8), *p* = 0.0064**

## Data Availability

All data presented is found in the publicly available databases listed in the Methods section.
